# Mechanisms of Infectivity and Evasion Derived from Microvesicles Cargo Produced by *Trypanosoma cruzi*

**DOI:** 10.3389/fcimb.2016.00161

**Published:** 2016-11-22

**Authors:** Bruna C. Borges, Isadora A. Uehara, Laysa O. S. Dias, Paula C. Brígido, Claudio V. da Silva, Marcelo J. B. Silva

**Affiliations:** ^1^Laboratório de Osteoimunologia e Imunologia dos Tumores, Instituto de Ciências Biomédicas, Universidade Federal de UberlândiaUberlândia, Brazil; ^2^Laboratório de Tripanossomatídeos, Instituto de Ciências Biomédicas, Universidade Federal de UberlândiaUberlândia, Brazil

**Keywords:** exosomes, microvesicles, *Trypanosoma cruzi*, cell invasion, cell evasion

## Abstract

Cell invasion by the intracellular protozoans requires interaction of proteins from both the host and the parasite. Many parasites establish chronic infections, showing they have the potential to escape the immune system; for example, *Trypanosoma cruzi* is an intracellular parasite that causes Chagas disease. Parasite internalization into host cell requires secreted and surface molecules, such as microvesicles. The release of microvesicles and other vesicles, such as exosomes, by different eukaryotic organisms was first observed in the late twentieth century. The characterization and function of these vesicles have recently been the focus of several investigations. In this review, we discuss the release of microvesicles by *T. cruzi*. The molecular content of these vesicles is composed of several molecules that take place during parasite-host cell interaction and contribute to the parasite-driven mechanism of evasion from the host immune system. These new findings appear to have a profound impact on the comprehension of *T. cruzi* biology and highlight novel potential strategies for developing more efficient therapeutic approaches.

Key points

- Exosomes cargo promotes Trypanosoma cruzi cell invasion and evasion from host immune response.
- Exosomes cargo derived from Trypanosoma cruzi modulates the content of host cell-derived exosomes.

## Introduction

Chagas' disease is an anthropozoic vector-borne parasitic infection, caused by the protozoan parasite, *Trypanosoma cruzi*. There are about six to seven million people infected in Latin America and approximately 25 million people living in areas with potential risk for infection. About 10,000 people die from the disease each year worldwide (WHO, 2015). The migration of infected individuals to non-endemic countries turned Chagas' disease into an emerging worldwide public health problem (Coura and Viñas, 2010; Álvarez et al., 2014).

*Trypanosoma cruzi* is a heterogeneous flagellate parasite, and its populations are characterized by a diverse morphology, heterogeneous biological behavior, high genetic variability, and distinctly different clinical courses (Macedo and Pena, 1998). The clonal-histotrophic model of Chagas' disease describes a correlation between the clonal-population structure of *T. cruzi* and its tissue tropism, and it explains the variety shown by this parasite (Macedo et al., 2004). It is now accepted that *T. cruzi* strains can be divided into six discrete typing units (DTUs), *T. cruzi* I to VI (Zingales et al., 2009).

Exosomes are formed within the endolysosomal network. The generation is initiated upon the endocytosis of extracellular material in the early endosome that results in multivesicular body (MVBs) formation. This compartment, also termed late endosome, has various intraluminal vesicles, which may degrade the cargo content or be secreted to the extracellular milieu as exosomes (Harding et al., 1983; Pan et al., 1985; Mantel and Marti, 2014). Exosomes contain specific proteins involved in vesicle formation and specific markers of the endosomal pathway, such as members of the Rab GTPase family, chaperones and tetraspanins (Ostrowski et al., 2010). Some have suggested extracellular vesicles (EVs) have a role in disease outcome, such as cancer, and physiological regulation because several infectious conditions lead to an increase in EVs in the body fluids of patients (Minciacchi et al., 2015). Some pathogenic organisms are capable of releasing exosomes, for example, the fungus *Cryptococcus neoformans* (Rodrigues et al., 2008), *Leishmania major* (Silvermann et al., 2010), and *Trypanosoma cruzi* (Bayer-Santos et al., 2013). EVs from eukaryotic parasites can be secreted from extracellular pathogens or produced by host cells infected by intracellular pathogens (Twu and Johnson, 2014). The EVs can mediate parasite-parasite and host-parasite interactions. Infected cell-derived exosomes enable communication between distant parasites and facilitate the spreading of virulence factors (Mantel and Marti, 2014).

Some authors have shown plasma membrane and flagellar pocket from *T.cruzi* epimastigote forms release vesicles, which comprise glycoproteins, glycolipids, and glycopeptides of the parasite surface (Da Silveira et al., 1979). *T. cruzi can* produce exosomes that stimulate host cells to produce EVs, in particular monocytes and lymphocytes, to modulate the host immune response (Cestari et al., 2012; Mantel and Marti, 2014). Later, in this sense, authors have found trypomastigote produces exosomes that contain surface components, like glycoproteins, such as gp85/transialidases, alphaGal-containing molecules, proteases, cytoskeleton proteins, mucins, and associated to GPI (glycosylphospatidylinositol)-anchored molecules. Besides proteins, small RNA in exosomes from *T. cruzi* has been reported, including tRNA, which were actively secreted to the extracellular medium and acted as vehicles for transferring these molecules to other parasites and to mammalian cells (Bayer-Santos et al., 2014).

Based on these results, intriguing questions may be raised: Why does the parasite shed these components? Would these exosomes play any role in the pathogenesis of Chagas disease or the evasion of parasite from the immune system? How would they modulate infection? These are the main topics of this article.

## Invasion mechanism of *Trypanosoma cruzi*

In the past decades, many laboratories have attempted to identify surface and secreted components of *T. cruzi* implicated in host-cell invasion, which consists of a multi-step process involving various parasite and host-cell molecules. To invade mammalian cells, some surface glycoproteins present in metacyclic trypomastigotes, such as gp82, gp35/50 or gp30, known as a gp82 variant expressed in gp82-deficient isolates, trigger events that lead to intracellular Ca^2+^ mobilization in both parasite and host cell (Burleigh and Andrews, 1998; Yoshida and Cortez, 2008). These parasites may also take advantage of secreted components, such as proteins from the SAP (serine-, alanine and proline-rich proteins) family; these proteins have a central domain (SAP-CD) responsible for invasion of mammalian cells by metacyclic forms (Baida et al., 2006; Zanforlin et al., 2013). Tissue culture-derived trypomastigotes (TCTs) have components, such as Tc-85, gp83, Tc-1, cruzipain, oligopeptidase B, and POP Tc80, that traverse the extracellular matrix and invade host cells (Burleigh and Andrews, 1998; Yoshida and Cortez, 2008). Through the surface molecules of gp85/transialidase superfamily, the parasites bind to fibronectin/laminin (Ouaissi et al., 1986; Giordano et al., 1994) and pave the way for the action of enzymes, such as the serine protease POP Tc80 that hydrolyses collagen (Santana et al., 1997; Grellier et al., 2001). Upon encountering the target cells, trypomastigotes attach to them in a manner mediated by Tc-85 (Alves et al., 1986), gp 83 (Villalta et al., 2001), or Tc-1 (Augustine et al., 2006). This interaction induces the activation of oligopeptidase B (Burleigh and Andrews, 1995; Caler et al., 1998) that generates a calcium-signaling factor from a precursor molecule. Alternatively, or simultaneously, cruzipain is secreted by attached trypomastigotes within the confines of parasite-target cell juxtaposition. Its action on the kininogen generates bradykinin, which interacts with its receptor and induces a calcium response (Scharfstein et al., 2000; Todorov et al., 2003). Calcium mobilization by all these factors inside the host cell promotes invasion. During this process, gp83 is released and can induce upregulation of laminin γ-1 expression by host cell (Kuratomi et al., 2002; Nde et al., 2006). Human galectin-3 enhances parasite adhesion to laminin (Moody et al., 2000), contributing to the pathogenesis of the disease.

After that, the protein P21 can be secreted in the parasite-target cell juxtaposition and activates a signaling cascade still unknown that leads calcium mobilization from parasite acidocalcisomes, phosphorylation of *T. cruzi* polypeptides (Fernandes et al., 2006), culminating in the activation of Rac1 and in membrane actin ruffles formation (Fernandes and Mortara, 2004) within microdomains enriched in cholesterol and GM1 (Fernandes et al., 2007) leading to parasite internalization. The protein P21 triggers host cell invasion by all infective forms. This protein binds to CXCR-4 receptor (Silva et al., 2009; Rodrigues et al., 2012), recruits immune cells, induces IL-4 production, and decreases blood vessels formation. This protein can be a potential target for developing novel treatment against chagasic cardiomyophaty (Teixeira et al., 2015).

During an *in vivo* infection, trypomastigotes present in the bloodstream can transform into extracellular amastigotes (EA), and when infected cells are ruptured, they may release intracellular amastigotes (IA). These amastigotes (EA and IA) may contribute to the infection progression by infecting cells in an actin-dependent manner, in a microenvironment context (Procópio et al., 1998, 1999). Amastigotes take advantage of carbohydrate epitopes present in Ssp-4 to attach to host cells prior to invasion (Silva et al., 2006).

The general expression of these proteins offers a potential target for novel therapies; besides inhibition of crucial biological pathways for parasite survival, the infective form appears to be a prospective strategy in specific drug discovery.

## Modulation mechanisms of extracellular vesicles cargo

Approximately 367 distinct proteins were identified as cargo of secreted vesicles and exosomes produced by *T. cruzi*. These proteomic analyses classified those proteins into 16 categories, involving host-parasite interaction, signaling, transporters, oxidation-reduction, carbohydrate metabolism, and others (Bayer-Santos et al., 2013).

**Figure 1:**
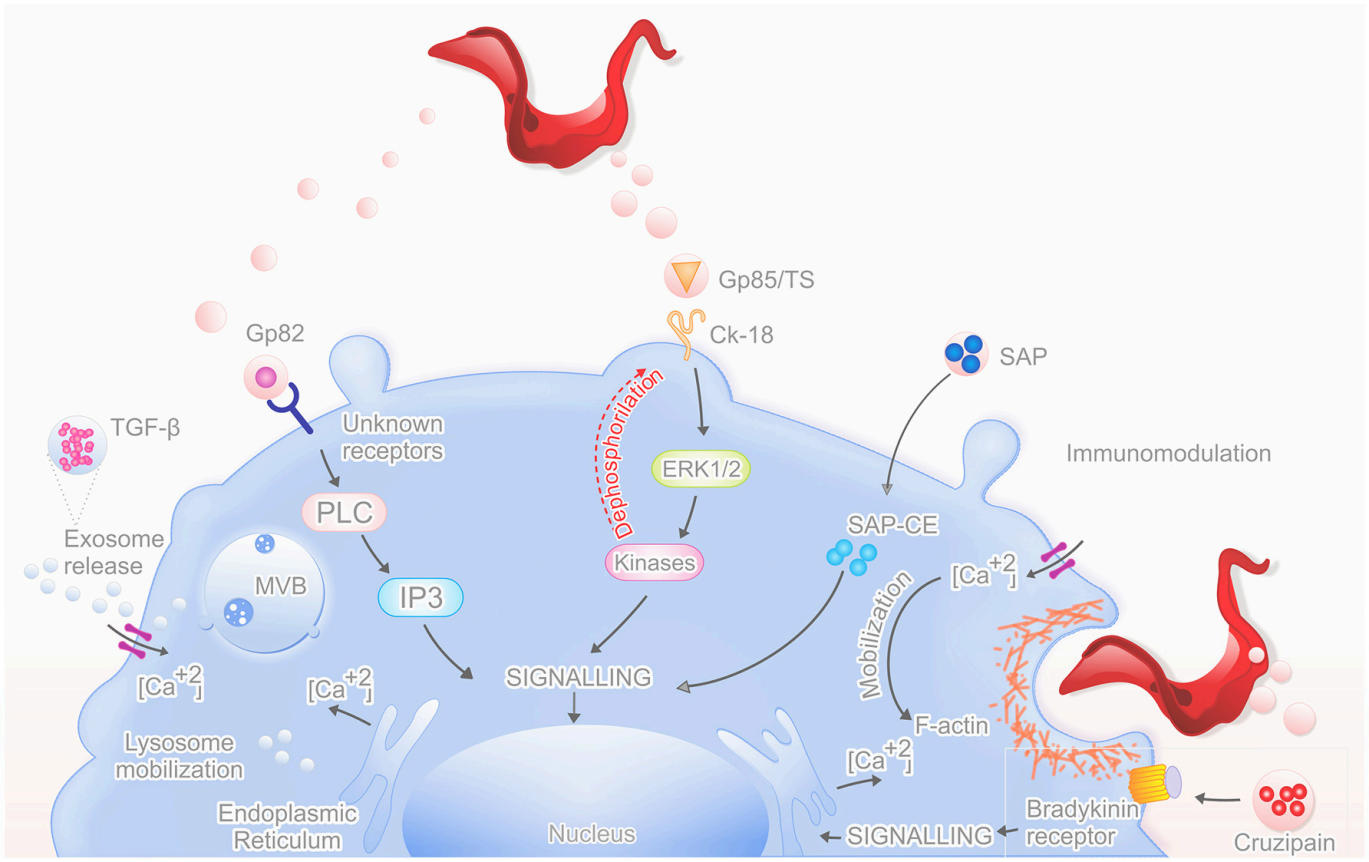
**Mechanism of action by secreted proteins in vesicles**. The graphical abstract shows the main proteins that are released in vesicles (TGF-β, Gp82, Gp85/TS, SAP, Cruzipain) and their mechanisms of action, which increases the parasite invasion of the host cell.

To invade host cells, *T. cruzi* metacyclic trypomastigote forms use distinct sets of surface and secreted molecules that interact with the host cell; some were found as cargo of microvesicles. In the past, different biological functions have been attributed to EVs (Denzer et al., 2000). Today, it is accepted that EVs represent an essential machinery for intercellular communication. Virulence factors delivery via vesicles cargo offers a potential mechanism to affect the host cells compared to simple diffusion (Mashburn-Warren and Whiteley, 2006; Rodrigues et al., 2008; Figure 1).

Trypanosome-secreted vesicles are associated with an inner plasma membrane leaflet protein, like flagellar calcium-binding protein (FCaBP), a-galactosyl glycoconjugates, GP35/50, and gp85/trans-sialidase superfamily (Trocoli Torrecilhas et al., 2009; Bayer-Santos et al., 2013). These compounds are important in parasite adhesion to host cells and probably have similar functions when located on secreted vesicles (Yoshida and Cortez, 2008). Also, inside extracellular vesicles, there are components with acid and alkaline phosphatase activities responsible for increased parasite adhesion and infection (Neves et al., 2014).

The nutrient starvation in epimastigotes induces the secretion of extracellular vesicles carrying small tRNAs and TcPIWI-tryp proteins as cargo. This cargo could be transferred to other parasites and to mammalian cells, increasing metacyclogenesis, and susceptibility of mammalian cells to infection (Fernandez-Calero et al., 2015).

Molecules secreted by *T. cruzi* into the extracellular medium may also participate in parasite internalization. The cruzipain is main cysteine protease of the *T. cruzi* and is present in all stages of the parasite, more abundant in replicating forms. In epimastigote, it is found in organelles similar to lysosome, the “reservosomes,” specific organelles pre-lysosomal-epimastigote, the reservosomes that are exocytic fusion of MVBs, resulting in exosomes (Bayer-Santos et al., 2013; Zanforlin et al., 2013). In amastigotes, cruzipain is associated with the plasma membrane through a GPI anchor. Other isoforms of this protein are secreted into the medium by trypomastigotes, which contributes to the virulence factor of Chagas disease (Santos, 2010; Alvarez et al., 2012). Cruzipain matures in the Golgi apparatus and is reserved in reservosomes, where it is also active. It promotes the penetration of trypomastigotes in host cells and plays an important role in the intracellular development and metacyclogenesis, in development of host immune response, and in the interaction with the insect host (Santos, 2010; Ferrão et al., 2015). This enzyme also contributes to the parasite's invasion in mammalian cells through the proteolysis of high and low molecular weight kininogen or by the activation of the cascading plasma prekallikrein conversion for active kallikrein (acting as kininogenases), then it in occurs the production of proinflammatory peptide (Lys-bradykinin), which interacts with receptors B2 (bradykinin receptor), G protein-coupled, inducing the increase of transient intracellular free calcium (Ca^2+^) (Del Nery et al., 1997; Scharfstein et al., 2000; Santos, 2010; Alvarez et al., 2012). The elevation of intracellular Ca^2+^ is one of the parasite invasion pathways leading to synaptotagmin VII-dependent migration and lysosome fusion to parasite binding site, preceding the formation of the parasitophorous vacuole (Aparicio et al., 2004). Cruzipain also works as an escape mechanism of the host immune response, because it makes digestion of “hinges” off all humans IgG subclasses (Berasain et al., 2003).

The contents of extracellular vesicles are enriched with glycoproteins of the gp85/trans-sialidase (TS) superfamily and other a-galactosyl (a-Gal) containing glycoconjugates, such as mucins; these proteins are released in different amounts, which may be determinant in the immunopathologic events not only in the early steps of infection, but also in the chronic phase (Nogueira et al., 2015). GP85/trans-sialidase has been shown as a conserved sequence or motif, called FLY, localized upstream to the C-terminal of the gp85/TS molecules. It promotes cell adhesion by binding to the intermediate filament protein cytokeratin-18 (CK18), which allows for dephosphorylation and activates ERK1/2 (extracellular signal regulated kinase) signaling cascade. This ERK1/2 cascade increases parasite entry into mammalian cells because these kinases are important for FLY binding to host cell, and consequently, in dephosphorylation of CK18 inducing redistribution and disassembly of filaments (Magdesian et al., 2006). This interaction is not limited to cytokeratin 18 (CK18), which may explain the wide variety of cells infected by the parasite (Tonelli et al., 2010).

GP82 is a cell adhesion molecule that binds to the host cell and is implicated in host-cell invasion of highly infective *T. cruzi* strains (Ramirez et al., 1993). The signaling cascade induced by gp82 when it binds to unknown receptors includes the participation of phospholipase C (PLC), which generates inositol triphosphate (IP_3_), which do Ca^2+^ mobilization from endoplasmic reticulum (Yoshida et al., 2000; Yoshida, 2006). This increase of intracellular Ca^2+^ leads to a rapid and transient reorganization of host cell microfilaments, including disassembly of the actin cytoskeleton facilitating the invasion by parasites (Dorta et al., 1995; Rodríguez et al., 1996; Ruiz et al., 1998; Martins et al., 2011).

SAP proteins are released into the extracellular medium by epimastigotes and metacyclic trypomastigotes as soluble factors or as components of secreted vesicles. The SAP-CD has regions SAP-NT (amino-terminal), SAP-CE (central), and SAP-CT (carboxy-terminal) (Baida et al., 2006). The interaction of SAP-CE fragment with host cells induce lysosome exocytosis by up-regulating calcium, probably acting synergistically with GP82 (Zanforlin et al., 2013). The lysosome exocytosis contributes to the formation of parasitophorous vacuoles (Martins et al., 2011).

*Trypanosoma cruzi* induce the release of exosomes from the cells they infect, such as monocytes and lymphocytes; these EVs also contain immunomodulatory cytokines, as TGF-β. This cytokine bearing this MVBs promoting enhance *T. cruzi* invasion leading to maturation and the continuation of the life cycle, contributing to escape the complement attack, a fact demonstrated by the inhibition of TGF-β by antibodies and receptor antagonists (Cestari et al., 2012) (Table 1).

**Table 1:** **Microvesicles cargo and respective function**.

**Components**	**Function**	**References**
- FCaBP - α-galactosyl glycoconjugates - GP35/50	Host cell parasite adhesion.	Yoshida and Cortez, 2008; Trocoli Torrecilhas et al., 2009; Bayer-Santos et al., 2013; Neves et al., 2014
- Small tRNAs - TcPIWI-tryp	Metacyclogenesis and susceptibility to infection.	Fernandez-Calero et al., 2015
- Cruzipain	Parasite host cell invasion. Digestion of “hinges” off all humans IgG subclasses.	Del Nery et al., 1997; Scharfstein et al., 2000; Berasain et al., 2003; Aparicio et al., 2004; Santos, 2010; Alvarez et al., 2012; Bayer-Santos et al., 2013; Zanforlin et al., 2013; Ferrão et al., 2015
- GP85/trans-sialidase superfamily	Cell adhesion, ERK1/2 activation.	Magdesian et al., 2006; Tonelli et al., 2010; Nogueira et al., 2015
- GP82	Host cell phospholipase C (PLC).	Ramirez et al., 1993; Dorta et al., 1995; Rodríguez et al., 1996; Ruiz et al., 1998; Yoshida et al., 2000; Yoshida, 2006; Martins et al., 2011
- SAP proteins	Cell adhesion, lysosome exocytosis, calcium influx.	Baida et al., 2006; Martins et al., 2011; Zanforlin et al., 2013
- TGF-β	*T. cruzi* invasion, contributing to escape the complement attack.	Cestari et al., 2012

## Exosomes and *Trypanosoma cruzi* infection: do they have any impact?

Antigens released via EVs are present at the parasite membrane and in the flagellar pocket before secretion by the trypomastigote (Gonçalves et al., 1991). Trocoli Torrecilhas and contributors have suggested a potential role of EVs in increase virulence by injecting them in BALB/C mice prior to infection with *Trypanosoma cruzi*, which leads to increase parasitaemia, and severe heart pathology and intense inflammation (Trocoli Torrecilhas et al., 2009). *T. cruzi* trypomastigotes produce their own EVs and can induce the production of EVs from other cells; such EVs can bind to trypomastigotes protecting them against lysis by the complement system (Cestari et al., 2012).

Both trypomastigote present in human blood and epimastigote present in the insect vector release EVs from the plasma membrane and from MVBs localized at the flagellar pocket (Bayer-Santos et al., 2013). Fractionation provided purified preparations of three fractions: the first enriched in larger vesicles resembling ectosomes, the second enriched in smaller vesicles resembling exosomes, and a third fraction enriched in soluble proteins not associated with extracellular vesicles. These data demonstrate a rich collection of proteins involved in the metabolism, signaling, nucleic acid binding, and parasite survival and virulence. These findings support the notion that *T. cruzi* uses different secretion pathways to excrete/secrete proteins (Bayer-Santos et al., 2013).

The release of exosomes leads to an increase in IL-4 and IL-10 secretion observed with a reduction in iNOS expression in CD4+ T cells and macrophages. This mechanism inducesTh2 immune response polarization causing dissemination and enhanced parasite survival (Trocoli Torrecilhas et al., 2009; Coakley et al., 2015).

Taken together, these data suggest *T. cruzi*-derived exosomes may play an important role in the invasion of the host-cell and the modulation of infection, favoring their perpetuation in the host. However, the mechanistic machinery behind this activity and the actual magnitude of the modulatory activity of these vesicles on mammalian host infection are still unclear.

## Author contributions

All authors participated in the write of the article. PB made the picture. BB, CS, and MS corrected the manuscript.

## Funding

The authors thank to Brazilian funding agencies FAPEMIG, CNPq, and CAPES which funded this publication, grants and scholarships.

### Conflict of interest statement

The authors declare that the research was conducted in the absence of any commercial or financial relationships that could be construed as a potential conflict of interest.
